# Prevalence and Interplay of Modifiable and Genetic Determinants of Eustachian Tube Dysfunction Among Saudi Adults: A Nationwide Study

**DOI:** 10.3390/diagnostics16010086

**Published:** 2025-12-26

**Authors:** Mohammad A. Jareebi, Riyadh A. Jahlan, Abdulrahman A. Otaif, Abdulelah A. Otaif, Abdulrahman A. Daghreeri, Mashael S. Mahnashi, Raghad W. Al Nahwe, Yahya A. Maslamani, Ali Y. Mashragi, Abdullah Mawkili, Wedad Mawkili, Faisal Hakami, Sulaiman Ahmed Hussain Darbashi, Majed A. Ryani, Ahmed A. Bahri

**Affiliations:** 1 Department of Family and Community Medicine, Faculty of Medicine, Jazan University, Jazan 45142, Saudi Arabia; majedryani@gmail.com (M.A.R.); dr.bahri2010@gmail.com (A.A.B.); 2 Faculty of Medicine, Jazan University, Jazan 45142, Saudi Arabia; rjahlan770@gmail.com (R.A.J.); otaifd7@gmail.com (A.A.O.); 3bdulelahotaif@gmail.com (A.A.O.); abdulrhmandaghriri@gmail.com (A.A.D.); 3 Department of Otolaryngology-Head and Neck Surgery, King Saud University Medical City, Riyadh 11461, Saudi Arabia; mashael2468m@gmail.com (M.S.M.); alnahwe.raghad@gmail.com (R.W.A.N.); 4 Public Health Authority–Southern Sector, Jazan 45142, Saudi Arabia; dr.yahyams@gmail.com; 5 Department of Otorhinolaryngology-Head and Neck Surgery, Jazan Health Cluster, Jazan 45142, Saudi Arabia; alimashragi@hotmail.com (A.Y.M.); faisalhakami1420@gmail.com (F.H.); 6 Department of Surgery, Faculty of Medicine, Jazan University, Jazan 45142, Saudi Arabia; mawkili@hotmail.com; 7 Department of Pharmacology and Toxicology, College of Pharmacy, Jazan University, Jazan 45142, Saudi Arabia; wmawkili@jazanu.edu.sa; 8 Department of Otorhinolaryngology, Jazan General Hospital, Jazan Health Cluster, Jazan 45142, Saudi Arabia; susu.vic.2010@gmail.com

**Keywords:** eustachian tube dysfunction, ETDQ-7, prevalence, risk factors, body mass index, allergies, hearing loss, Saudi Arabia

## Abstract

**Background/Objectives**: Eustachian Tube Dysfunction (ETD) is a prevalent condition affecting middle ear pressure regulation, yet nationwide epidemiological data in Saudi Arabia remain limited. This study aimed to assess the prevalence of ETD and identify its associated factors among Saudi adults using a validated screening tool. **Methods**: A nationwide cross-sectional study was conducted between June 2024 and March 2025 among 1124 Saudi adults (aged ≥ 18 years) across five geographic regions. ETD was assessed using the validated Arabic version of the seven-item Eustachian Tube Dysfunction Questionnaire (ETDQ-7), with scores ≥ 14.5 indicating dysfunction. Data on demographic, anthropometric, clinical, and lifestyle characteristics were collected via an online questionnaire. Multiple linear regression analysis identified independent predictors of ETDQ-7 scores, with statistical significance set at *p* < 0.05. **Results**: The prevalence of ETD was 33.9% (95% CI: 31.1–36.8%), substantially higher than the 7% self-reported rate. Of affected participants, 29.6% had mild-to-moderate ETD and 4.3% had severe dysfunction. Multivariable regression analysis identified four significant independent predictors: higher body mass index (BMI) (β = 0.08; 95% CI: 0.03–0.16; *p* = 0.049), family history of hearing loss (β = 1.87; 95% CI: 0.90–2.83; *p* < 0.001), prior bariatric bypass surgery (β = 14.37; 95% CI: 3.33–25.41; *p* = 0.011), and allergies (β = 3.19; 95% CI: 2.30–4.07; *p* < 0.001). No significant associations were found with demographic factors, smoking, or other comorbidities. **Conclusions**: ETD affects approximately one-third of Saudi adults, with significant underdiagnosis. Obesity, genetic predisposition, bariatric surgery, and allergic conditions represent key modifiable and non-modifiable risk factors. These findings support implementing routine ETDQ-7 screening in primary care and targeted interventions for high-risk populations.

## 1. Introduction

The Eustachian tube (ET) is a narrow anatomical conduit connecting the middle ear to the nasopharynx, serving three essential physiological functions: regulation of middle ear pressure, ventilation of the tympanic cavity, and clearance of secretions through mucociliary action [1,2]. Additionally, it prevents the reflux of nasopharyngeal secretions and sound into the middle ear [1]. When these functions become impaired, the resulting condition is termed Eustachian Tube Dysfunction (ETD) [3]. ETD represents a common otological disorder that predisposes individuals to various middle ear pathologies, including otitis media with effusion (OME), tympanic membrane atelectasis, and cholesteatoma, potentially leading to conductive hearing loss and significant morbidity [4,5]. Globally, ETD affects approximately 5% of adults and up to 40% of children, with transient dysfunction being particularly common during early developmental stages [6,7]. In the United States alone, ETD accounts for over 2 million annual outpatient visits among adults aged 20 years and older, reflecting its substantial clinical and economic burden [3]. Despite its high prevalence, ETD remains underdiagnosed due to nonspecific symptoms such as aural fullness, muffled hearing, and tinnitus, coupled with the historical absence of standardized diagnostic criteria [8]. This diagnostic gap often delays appropriate treatment, increasing the risk of complications. Untreated ETD progresses to OME in 30–40% of cases, chronic tympanic membrane retraction in 15–25%, and cholesteatoma development in 5–10% of patients with long-standing dysfunction [9]. The seven-item Eustachian Tube Dysfunction Questionnaire (ETDQ-7) addresses this diagnostic challenge by providing a validated, symptom-based assessment tool that facilitates early detection, monitors treatment response, and quantifies symptom burden [10]. Each item is rated on a seven-point Likert scale, yielding scores ranging from 7 to 49, with scores ≥ 14.5 indicating clinically significant dysfunction [10,11]. The ETDQ-7 has demonstrated excellent reliability (Cronbach’s alpha > 0.9) and has been validated in multiple languages including Arabic [12]. However, certain limitations merit consideration, including potential interpretation challenges for low-literacy populations and the absence of differentiation between acute and chronic ETD [10]. Regional studies in Saudi Arabia have reported variable ETD prevalence rates: 42.5% in Jeddah [13], 41.3% in Al-Madinah [14], 21.1% in Taif [15], and 12.2% in Qassim [16]. These disparities likely reflect differences in sampling methodologies, population characteristics, and environmental factors. Key risk factors identified include environmental exposures, allergic conditions, anatomical anomalies, and recurrent upper respiratory infections—all of which impair mucociliary function and tubal patency [17,18]. Environmental pollution represents a particularly relevant concern in Saudi Arabia, where major cities exceed World Health Organization (WHO) PM2.5 guidelines. Riyadh and Jeddah record PM2.5 levels of 12.8 μg/m^3^ and 12.0 μg/m^3^, respectively, substantially above the WHO annual mean recommendation of 5 μg/m^3^ [19,20]. Additionally, smoking prevalence remains significant in Saudi Arabia, with rates of 21.3% in Jeddah and 18.7% in Riyadh [21]. Furthermore, approximately 70% of patients with chronic middle ear conditions report concurrent ETD symptoms, highlighting the interconnection between ETD and severe otological pathology [22].

Despite the above findings, comprehensive regional data on ETD in the Saudi population remain limited, restricting a clear understanding of the true population burden and the relative contribution of associated risk factors. To address this gap, the present study aimed to (1) determine the nationwide prevalence of ETD among Saudi adult population using the ETDQ-7, (2) identify demographic, clinical, and lifestyle factors associated with ETD, and (3) compare the consistency between subjective ETDQ-7 scores and general subjective symptom reporting to assess diagnostic awareness. It is hypothesized that demographic, clinical, and lifestyle factors are significantly linked with ETD prevalence, and that ETDQ-7 scores may correlate with patients’ self-reported symptoms, reflecting reliable diagnostic awareness. This approach offers novel insights compared with previous regional studies and may help in informed healthcare planning, screening, and preventive strategies for high-risk populations.

## 2. Materials and Methods

### 2.1. Study Design and Setting

This cross-sectional study was conducted across all five geographic regions of Saudi Arabia (Central, Eastern, Western, Northern, and Southern) between September 2024 and March 2025. The study employed a convenience sampling approach with strategic efforts to ensure broad geographic and demographic representation through multi-channel recruitment.

### 2.2. Participants and Sampling

The target population comprised Saudi adults aged ≥18 years residing in Saudi Arabia. Inclusion criteria required participants to be at least 18 years old, provide informed consent, and complete at least 90% of the questionnaire items. Exclusion criteria included individuals under 18 years, those unable to provide consent, and participants with >10% incomplete responses. Sample size calculation was based on the estimated Saudi population of 35 million with approximately 65% adults (22.75 million) [23]. Using Raosoft software with an expected ETD prevalence of 50% (to maximize sample size), 95% confidence level, 5% margin of error, and accounting for 10% non-response rate, the minimum required sample was 385 participants. To enhance statistical power for subgroup analyses and improve representativeness across diverse demographic strata, the target sample was increased to 1124 participants. Recruitment utilized multiple channels to maximize reach and minimize selection bias: digital dissemination through social media platforms (WhatsApp, Twitter/X). The monitoring of the geographical depiction of the sample population was conducted by obtaining self-reported geographical data from the survey participants at the time of survey completion. The number of responses from each geographical area was tracked and evenly distributed to ascertain equitable geographical representation. Finally, demographic data and geographical region were analyzed simultaneously to maintain both diversity and proportional representation within the study cohort.

### 2.3. Data Collection Instrument

Data was collected using a validated, structured Arabic-language questionnaire adapted from previous research [13]. The questionnaire comprised four sections:

Section 1 (Sociodemographic characteristics): Twelve items assessing age, gender, nationality, region of residence, urban versus rural setting, nature of residence (coastal/mountainous/plain), educational level, employment status, marital status, and monthly income in Saudi Riyals (SAR).

Section 2 (Anthropometric and general health): Eight items evaluating self-reported weight, height, body mass index (BMI), physical activity level (categorized as none, irregular [<30 min five times weekly], or regular [≥30 min five times weekly]), smoking status (never, current, former), and physician-diagnosed diabetes mellitus, hypertension, and heart disease.

Section 3 (Clinical and surgical history): Six items documenting personal and family history of hearing loss, history and type of weight-loss surgery (bypass or gastric sleeve), presence of allergies, and specific allergic conditions (allergic rhinitis, asthma, gastroesophageal reflux disease).

Section 4 (ETD assessment): The validated Arabic version of the ETDQ-7 [12] alongside questions regarding self-reported ETD diagnosis. The ETDQ-7 translation followed rigorous forward–backward translation methodology with expert review to ensure cultural and content validity [12]. Each of the seven ETDQ-7 items was rated on a seven-point Likert scale (1 = no problem to 7 = severe problem), yielding total scores ranging from 7 to 49. A mean item score ≥ 2.5 or total score ≥ 14.5 was considered indicative of ETD, consistent with established diagnostic thresholds [10,11]. Severity classification categorized participants as normal (ETDQ-7 < 14.5), mild-to-moderate (14.5–24.9), or severe (≥25) [10]. Pilot testing with 30 participants confirmed good reliability (Cronbach’s alpha = 0.87), consistent with the established internal consistency of ETDQ-7 (Cronbach’s alpha > 0.9) [10,12].

### 2.4. Statistical Analysis

Following data collection, responses were exported to Microsoft Excel (Microsoft Corporation, Redmond, WA, USA) for data cleaning, error checking, duplicate removal, and preliminary screening for completeness. All statistical analyses were performed using R software (version 4.2.3; R Foundation for Statistical Computing, Vienna, Austria). Descriptive statistics summarized participant characteristics using means and standard deviations for continuous variables and frequencies with percentages for categorical variables. ETD prevalence was calculated as the proportion of participants with ETDQ-7 scores ≥ 14.5, with 95% confidence intervals. Stratified prevalence estimates were computed across demographic and clinical subgroups, with chi-square tests assessing between-group differences.

Multiple linear regression analysis identified independent predictors of ETDQ-7 scores (treated as a continuous outcome variable). All predictor variables were entered simultaneously into the model to control for potential confounding. Model fit was evaluated using R^2^, adjusted R^2^, and F-statistics. A sensitivity analysis excluding participants with bypass surgery was conducted to assess the robustness of other predictors. Statistical significance was set at *p* < 0.05 for all analyses. The completeness of the questionnaire was assessed for each patient, and any missing responses were documented. Cases with incomplete data were removed during the analysis.

### 2.5. Ethical Considerations

The study protocol received approval from the Research Ethics Committee of Jazan University (REC-46/02/1166, approved 1 September 2024). The study adhered to the Declaration of Helsinki principles, with written informed consent obtained from all participants prior to enrollment. Participation was entirely voluntary and anonymous, with participants retaining the right to withdraw at any point without consequences. Data confidentiality and anonymity were strictly maintained throughout the study period, with secure data storage and access limited to authorized research personnel only.

## 3. Results

### 3.1. Sociodemographic Characteristics

The study included 1124 participants, predominantly female (69%) and Saudi nationals (94%), with a mean age of 32 ± 13 years. Geographically, 35% resided in the western region, 25% in the southern, 19% in the central, 12% in the eastern, and 9% in the northern regions. Most participants lived in urban areas (87%), with 52% inhabiting plains, 25% coastal zones, and 23% mountainous regions. Education levels varied: 59.9% held bachelor’s degrees or diplomas, 31.6% had high school education or less, and around 9% possessed master’s or doctoral qualifications. Employment status revealed 40% were employed, 39% were students, and 21% were unemployed. Marital status distributions included 52% unmarried or engaged, 44% married, and 4% divorced or widowed. Monthly income disparities were notable, with approximately 46% earning <5000 SAR, 18.6% earning 5000–9999 SAR, and 17.5% and 18.1% earning 10,000–14,999 SAR and ≥15,000 SAR, respectively (Table 1).

### 3.2. Anthropometric Measures and Health Characteristics

The participants had a mean BMI of 25 ± 5.9 kg/m^2^, with nearly half (48%) in the normal weight range and 45% overweight or obese. In terms of physical exercise, 28% met recommended activity levels. The majority were non-smokers (87%). Among comorbidities, 8% of patients reported that they had diabetes, while 10% had hypertension (Table 2).

### 3.3. Clinical and Surgical Factors Associated with ETD

Clinical histories revealed that 4% of participants had personal hearing loss, while 25% reported a family history of hearing loss. Weight-loss surgery was reported by 4.3% of participants, including 0.7% who had undergone bypass surgery and 3.6% who had undergone gastric sleeve surgery. Allergies were reported by 33.6% of participants, with the most common types being allergic rhinitis (10.5%), asthma (6.2%), and gastroesophageal reflux disease (GERD) (4.4%) (Table 3).

### 3.4. Prevalence of Eustachian Tube Dysfunction

**Figure 1:**
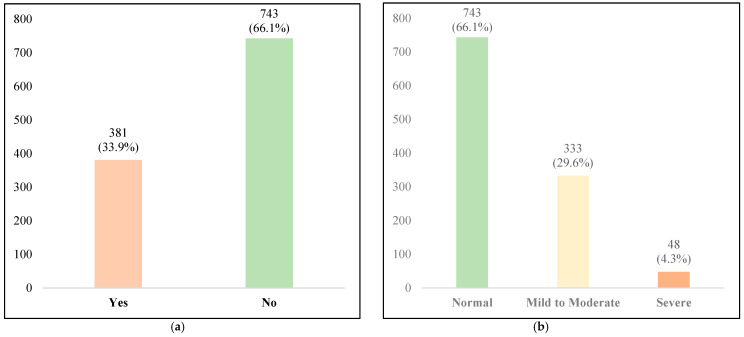
(**a**) Prevalence of eustachian tube dysfunction (ETD) among participants, showing that 33.9% screened positive while 66.1% screened negative. (**b**) Severity classification of ETD based on ETDQ-7 scores, demonstrating that 66.1% had normal function, 29.6% had mild to moderate dysfunction, and 4.3% had severe dysfunction.

Based on ETDQ-7 scores (mean: 13 ± 7.1), the overall prevalence of ETD was 33.9% (95% CI: 31.1–36.8%), with 381 participants meeting the diagnostic threshold (ETDQ-7 score ≥ 14.5). In contrast, only 7% (*n* = 84) of participants self-reported ETD-related symptoms, indicating substantial underdiagnosis. Severity stratification classified 66.1% (*n* = 743) as normal (ETDQ-7 score < 14.5), 29.6% (*n* = 333) as mild to moderate ETD (ETDQ-7 score 14.5–24.9), and 4.3% (*n* = 48) as severe ETD (ETDQ-7 score ≥ 25) (Table 4, Figure 1a,b).

### 3.5. ETD Prevalence Stratified by Key Characteristics

ETD prevalence varied significantly across BMI categories, with obese participants showing the highest rates (42.4%) compared to normal-weight individuals (29.9%; *p* = 0.003). Similarly, participants with allergies had substantially higher ETD prevalence (49.2%) compared to those without allergies (26.5%; *p* < 0.001). Parameters including gender, residence, region, physical activity and smoking status showed no significant differences (*p* > 0.05) (Table 5).

### 3.6. Predictors of ETD Severity

Multiple linear regression analysis identified several significant independent predictors of ETDQ-7 scores. Higher BMI was associated with elevated ETD scores (β = 0.08; 95% CI: 0.03–0.16; *p* = 0.049), translating to approximately a 0.4-point increase in ETDQ-7 score for every 5-unit BMI increase. Family history of hearing loss was strongly associated with higher ETD scores (β = 1.87; 95% CI: 0.90–2.83; *p* < 0.001), representing an increase of approximately 13% of one standard deviation in ETDQ-7 scores. Prior bypass surgery showed the largest effect size (β = 14.37; 95% CI: 3.33–25.41; *p* = 0.011); however, this finding should be exploratory and interpreted with caution given the small sample size (*n* = 8, 0.7% of cohort) and wide confidence interval. The presence of allergies emerged as a strong predictor, with allergy diagnosis associated with a 3.19-point increase in ETDQ-7 score (95% CI: 2.30–4.07; *p* < 0.001) (Table 6). Detailed findings are presented in Appendix A (Table A1).

### 3.7. Sensitivity Analysis

A sensitivity analysis excluding participants who underwent bypass surgery (*n* = 8) was conducted to assess the robustness of other predictors. The associations for BMI (β = 0.08, *p* = 0.051), family history of hearing loss (β = 1.85, *p* < 0.001), and allergies (β = 3.17, *p* < 0.001) remained essentially unchanged, confirming the stability of these findings. Model fit improved slightly (Adjusted R^2^ = 0.121).

## 4. Discussion

### 4.1. Principal Findings and Prevalence Context

This study identified an ETD prevalence of 33.9% (95% CI: 31.1–36.8%) among Saudi adults based on validated ETDQ-7 assessment, substantially exceeding the 7% self-reported rate and revealing a 4.8-fold diagnostic gap. Independent risk factors included higher BMI, family history of hearing loss, prior bariatric bypass surgery, and allergies, whereas demographic characteristics, smoking, and common comorbidities showed no significant associations. These findings establish ETD as a major underdiagnosed public health concern in Saudi Arabia, affecting approximately one in three adults.

The observed prevalence substantially exceeds rates reported in Western populations, including 12.5% in the United States [24] and 19.8% in Germany [25], but aligns closely with regional Madinah [26]. It is also notably higher than a study from the Aseer region, which re-ported a much lower ETD prevalence of 4% [14]. This geographic pattern likely reflects environmental factors (arid climate, dust exposure, elevated air pollution), higher allergy prevalence (30–40% versus 20–25% in Western populations), and elevated obesity rates (35% in Saudi Arabia versus 27% in the United States) [19,20,27,28].

### 4.2. The Diagnostic Awareness Gap

The discrepancy between ETDQ-7-based diagnosis (33.9%) and self-reported symptoms (7%) represents a critical finding with substantial clinical implications. This 4.8-fold under-recognition parallels international observations, including a Turkish study where 31.2% met ETDQ-7 criteria yet only 6.8% self-reported symptoms [29], and an earlier Saudi study where only 9% of ETDQ-7-positive cases recognized their symptoms as abnormal [13]. This consistent pattern of underdiagnosis likely stems from multiple factors: gradual symptom onset leading to normalization, attribution of symptoms to alternative causes such as sinusitis or seasonal allergies, limited health literacy regarding ETD, and the nonspecific nature of symptoms (ear fullness, muffled hearing). The clinical significance is substantial, as undiagnosed ETD increases the risk of progressive complications, including chronic OME, tympanic membrane retraction, and cholesteatoma formation [9]. These findings strongly support implementing systematic ETDQ-7 screening in primary care settings.

### 4.3. Obesity as a Modifiable Risk Factor

Our analysis revealed that each 1-unit BMI increase was associated with a 0.08-point elevation in ETDQ-7 score (*p* = 0.049), translating to approximately a 0.4-point increase per 5-unit BMI increment. Stratified analysis demonstrated a dose–response relationship, with ETD prevalence increasing from 29.9% in normal-weight individuals to 42.4% in obese participants (*p* = 0.003). These findings align with established international evidence, including a U.S. cohort study demonstrating 12% higher ETD prevalence per BMI unit [30] and a German investigation showing 9.7% increased risk per BMI unit with obese individuals exhibiting 2.3-fold higher risk compared to normal-weight counterparts [31].

The pathophysiological mechanisms linking obesity to ETD are multifactorial. Adipose tissue secretes pro-inflammatory cytokines including leptin and interleukin-6, which promote mucosal edema and inflammation within the Eustachian tube [32]. Additionally, increased fat deposition in the parapharyngeal space may cause mechanical compression of the tubal ostium, impairing ventilation. These findings identify weight management as a potentially effective intervention for ETD prevention and symptom amelioration in populations with elevated obesity rates.

### 4.4. Allergies and Inflammatory Mechanisms

Allergy diagnosis emerged as the strongest predictor of ETD severity, with allergic individuals showing a 3.19-point ETDQ-7 score increase (*p* < 0.001), representing approximately 45% of one standard deviation change. Stratified analysis revealed ETD prevalence of 49.2% among allergic participants versus 26.5% in non-allergic individuals (*p* < 0.001). These findings corroborate established literature demonstrating 4.2-fold higher ETD odds in allergic patients [33] and 3.8-fold increased odds specifically for allergic rhinitis sufferers. The mechanistic basis is well-established: allergen exposure triggers IgE-mediated mast cell degranulation, releasing histamine and leukotrienes that induce mucosal inflammation, edema, and increased mucus production within the Eustachian tube. Given Saudi Arabia’s high burden of allergic diseases, particularly allergic rhinitis affecting 30–40% of adults [27,28], aggressive allergy management represents a promising avenue for reducing ETD prevalence.

### 4.5. Bariatric Surgery: A Novel Association

Bariatric bypass surgery demonstrated the largest effect size in the regression model (β = 14.37; 95% CI: 3.33–25.41; *p* = 0.011), indicating a novel but preliminary association due to the small sample size (*n* = 8, 0.7% of cohort) and wide confidence interval. Gastric sleeve surgery showed a similar, non-significant trend (β = 5.57; *p* = 0.266). Sensitivity analysis excluding bypass cases confirmed the stability of other predictors. These findings should be interpreted cautiously and require validation in larger prospective studies with objective ET function testing before clinical recommendations.

### 4.6. Genetic Predisposition and Family History

Those with family histories of hearing loss showed markedly higher ETDQ-7 scores (β = 1.87; 95% CI: 0.90–2.83; *p* < 0.001). Additional analysis found an ETD prevalence of 44.6% in those with a family history, compared to 30.3% without (*p* = 0.002). Previous research has explored possible genetic links to middle-ear conditions through COL11A2-related hearing loss and heritable estimates from twin studies [17,18]. However, caution is needed when interpreting the role of family history, given possible influences from shared environments, specific otologic health, or multifactorial risks. Nonetheless, a family history of hearing loss does appear to place individuals in a higher risk group, which may require increased surveillance, earlier intervention, or ETD diagnostic testing.

### 4.7. Null Findings and Clinical Implication

Several anticipated associations were notably absent in our analysis. Smoking status showed no significant relationship with ETD (*p* = 0.436), contrasting with U.S. data demonstrating 2.1-fold higher ETD risk among smokers [10]. This discrepancy likely reflects our cohort’s substantially lower smoking prevalence (8% versus 15% nationally in the U.S.) and younger mean age (32 versus 45 years in comparative studies), potentially limiting statistical power to detect this association. Similarly, no regional variation in ETD prevalence emerged (*p* = 0.189), despite documented differences in air quality across Saudi cities [19,20]. This unexpected finding may result from our predominantly urban sample (87%) obscuring geographic differences, or suggest that individual-level factors (BMI, allergies) exert stronger influence than environmental exposures in determining ETD risk. Furthermore, no significant associations were observed with age, gender, region of residence, nature of residence (coastal/mountainous/plain), education level, income, marital status, employment, smoking status, physical activity level, or comorbidities including diabetes mellitus, hypertension, or heart disease. This may reflect the low prevalence of these conditions in the cohort, limited statistical power, or the predominance of stronger predictors in the regression model, rather than a definitive absence of effects.

### 4.8. Strengths and Limitations

This study possesses several strengths: it represents the largest and most geographically comprehensive investigation of ETD in Saudi Arabia with nationwide representation (*n* = 1124); uses the validated Arabic ETDQ-7 for standardized assessment; identifies the novel bariatric surgery–ETD association; achieves high response rate (93%); and provides adequate statistical power. However, certain limitations should be acknowledged. Because of the cross-sectional design, the temporal relationship between potential predictors and ETD cannot be determined, and thus no causal inference can be made. Convenience sampling introduced may cause selection bias, with overrepresentation of urban (87%), young (mean age 32), and female (69%) participants relative to national demographics. This sampling bias may limit the generalizability of our findings, as the attitudes and behaviors of younger, urban, and predominantly female participants may not reflect the broader population. Therefore, observed associations particularly those based on self-reported measures and the small bypass-surgery (*n* = 8) subgroup warrants cautious interpretation of the results. Self-reported anthropometric measures may introduce bias, leading to BMI misclassification and affecting observed associations. As the regression model’s R^2^ is low (0.14), it must be acknowledged that the predictors explain only a modest portion of the variance, suggesting that other unmeasured factors may play a substantial role. Future studies could address this by using objectively measured data or validating self-reports values of sample.

### 4.9. Clinical and Public Health Implications

These findings carry substantial implications for clinical practice and public health policy in Saudi Arabia. The high prevalence (33.9%) combined with marked underdiagnosis (4.8-fold gap) justifies implementing systematic ETDQ-7 screening in primary care settings, particularly for high-risk groups, including obese individuals, allergy sufferers, bariatric surgery patients, and those with family history of hearing loss. The identification of modifiable risk factors—particularly obesity and allergies—provides clear targets for preventive interventions. Weight management programs and aggressive allergy management may improve ETD symptoms and reduce prevalence. For healthcare providers, these findings emphasize comprehensive otological assessment in patients presenting with nonspecific auditory symptoms. Post-bariatric surgery patients warrant specific attention for ETD surveillance. Future research priorities should include prospective cohort studies establishing temporal relationships, mechanistic investigations, intervention trials evaluating weight loss and allergy treatment efficacy, and validation studies incorporating objective Eustachian tube function testing.

## 5. Conclusions

This regional study demonstrates ETD as a highly prevalent but underdiagnosed condition in the Saudi population, with significant gaps in symptom recognition. Key risk factors, such as obesity, allergies, bariatric surgery, and family history, suggest opportunities for targeted interventions, including routine ETDQ-7 screening, weight management, allergy control, post-bariatric follow-up, and public awareness campaigns. The observed association between bariatric surgery and ETD was preliminary, but it recommends further research and may inform standardized surgical counseling. Future investigation should focus on prospective studies and intervention trials to guide prevention and management strategies.

## Figures and Tables

**Table 1:** Sociodemographic characteristics of study participants (*n* = 1124).

Characteristic	Value
**Age, mean ± SD (years)**	32 ± 13
**Sex, *n* (%)**	
Male	344 (31%)
Female	780 (69%)
**Nationality, *n* (%)**	
Saudi	1052 (94%)
Non-Saudi	72 (6%)
**Region, *n* (%)**	
Central region	212 (19%)
Eastern region	134 (12%)
Northern region	102 (9%)
Southern region	279 (25%)
Western region	397 (35%)
**Residence, *n* (%)**	
Rural	143 (13%)
Urban	981 (87%)
**Nature of residence, *n* (%)**	
Coastal	278 (25%)
Mountainous	257 (23%)
Plain	589 (52%)
**Education level, *n* (%)**	
High School Degree or Lower	355 (31.6%)
Bachelor’s or Diploma degree	673 (59.9%)
Master’s or PhD	96 (8.5%)
**Employment status, *n* (%)**	
Employed	448 (40%)
Student	440 (39%)
Unemployed	236 (21%)
**Marital status, *n* (%)**	
Non-Married/Engaged	586 (52%)
Married	494 (44%)
Divorced/Widow	44 (4%)
**Monthly Income (SAR), *n* (%)**	
<5000	515 (45.8%)
5000–9999	209 (18.6%)
10,000–14,999	197 (17.5%)
≥15,000	203 (18.1%)

Note: SD = Standard deviation; SAR = Saudi Riyal.

**Table 2:** Anthropometric measures, lifestyle factors, and comorbidities of study participants (*n* = 1124).

Characteristic	Value
**Anthropometric Measures, mean ± SD**	
Weight (kg)	67 ± 17
Height (cm)	163 ± 8.8
BMI (kg/m²)	25 ± 5.9
**BMI Categories (WHO), *n* (%)**	
Underweight (<18.5)	79 (7%)
Normal weight (18.5–24.9)	539 (48%)
Overweight (25–29.9)	315 (28%)
Obese (≥30)	191 (17%)
**Physical activity level, *n* (%)**	
No regular activity	578 (51.4%)
Regular (≥30 min, 5×/week)	319 (28.4%)
Irregular (<30 min, 5×/week)	227 (20.2%)
**Smoking status, *n* (%)**	
Never	979 (87%)
Current smoker	88 (8%)
Former smoker	57 (5%)
**Comorbidities, *n* (%)**	
Diabetes Mellitus	
No	1036 (92%)
Yes	88 (8%)
Hypertension	
No	1012 (90%)
Yes	112 (10%)
Heart Disease	
No	1081 (96%)
Yes	43 (4%)

Note: SD = Standard deviation; BMI = Body mass index; WHO = World Health Organization.

**Table 3:** Clinical and surgical factors associated with eustachian tube dysfunction (*n* = 1124).

Characteristic	*n* (%) *
**Personal history of hearing loss**	
No	1083 (96%)
Yes	41 (4%)
**Family history of hearing loss**	
No	844 (75%)
Yes	280 (25%)
**History of weight-loss surgery**	
No	1076 (96%)
Yes	48 (4%)
**Type of weight-loss surgery (** *n* ** = 48)**	
Bypass surgery	8 (0.7%)
Gastric sleeve surgery	40 (3.6%)
**Presence of allergy**	
No	746 (66.4%)
Yes	378 (33.6%)
**Type of allergic condition (** ***n*** ** = 378)**	
Allergic rhinitis	118 (10.5%)
Asthma	70 (6.2%)
Gastroesophageal reflux disease (GERD)	49 (4.4%)
Multiple allergic conditions	141 (12.5%)

* Percentages for “Type of weight-loss surgery” and “Type of allergic condition” are calculated from total sample (*n* = 1124) for consistency.

**Table 4:** ETD diagnosis and severity classification (*n* = 1124).

Characteristic	Value
**ETDQ-7 Score**	
Mean ± SD	13 ± 7.1
**ETD Diagnosis (ETDQ-7 ≥ 14.5), *n* (%)**	
No	743 (66.1%)
Yes	381 (33.9%)
**Self-reported ETD symptoms, *n* (%)**	
No	1040 (93%)
Yes	84 (7%)
**ETD Severity Classification, *n* (%)**	
Normal (ETDQ-7 < 14.5)	743 (66.1%)
Mild to Moderate (ETDQ-7 14.5–24.9)	333 (29.6%)
Severe (ETDQ-7 ≥ 25)	48 (4.3%)
**Discordance Rate**	
ETDQ-7 * positive but self-report negative	297 (26.4%)
Self-report positive but ETDQ-7 negative	0 (0%)

* ETDQ-7 = Eustachian Tube Dysfunction Questionnaire-7.

**Table 5:** ETD prevalence stratified by demographic and clinical characteristics (*n* = 1124).

Characteristic	ETD Present *n* (%)	ETD Absent *n* (%)	*p*-Value *
**Overall Prevalence**	381 (33.9%)	743 (66.1%)	-
**Gender**			0.304
Male	109 (31.7%)	235 (68.3%)	
Female	272 (34.9%)	508 (65.1%)	
**Region**			0.189
Central	77 (36.3%)	135 (63.7%)	
Eastern	39 (29.1%)	95 (70.9%)	
Northern	30 (29.4%)	72 (70.6%)	
Southern	89 (31.9%)	190 (68.1%)	
Western	146 (36.8%)	251 (63.2%)	
**Residence**			0.413
Rural	44 (30.8%)	99 (69.2%)	
Urban	337 (34.4%)	644 (65.6%)	
**BMI Category (WHO)**			0.003
Underweight (<18.5)	22 (27.8%)	57 (72.2%)	
Normal weight (18.5–24.9)	161 (29.9%)	378 (70.1%)	
Overweight (25–29.9)	117 (37.1%)	198 (62.9%)	
Obese (≥30)	81 (42.4%)	110 (57.6%)	
**Allergy Status**			<0.001
No allergy	195 (26.5%)	541 (73.5%)	
Allergy present	186 (49.2%)	192 (50.8%)	
**Smoking Status**			0.281
Never	337 (34.4%)	642 (65.6%)	
Current smoker	25 (28.4%)	63 (71.6%)	
Former smoker	19 (33.3%)	38 (66.7%)	
**Physical Activity**			0.187
No regular activity	207 (35.8%)	371 (64.2%)	
Regular (≥30 min, 5×/week)	100 (31.3%)	219 (68.7%)	
Irregular (<30 min, 5×/week)	74 (32.6%)	153 (67.4%)	
**Family History of Hearing Loss**			0.002
No	256 (30.3%)	588 (69.7%)	
Yes	125 (44.6%)	155 (55.4%)	
**Weight-loss Surgery**			0.029
No	359 (33.4%)	717 (66.6%)	
Yes	22 (45.8%)	26 (54.2%)	

* *p*-values calculated using chi-square test for categorical variables.

**Table 6:** Significant determinants of ETD: Multiple linear regression analysis (*n* = 1124).

Predictors	β	95% CI	*p*-Value
**(Intercept)**	9.91	6.38–13.43	<0.001
**Anthropometric**			
BMI (per kg/m^2^)	0.08	0.03–0.16	0.049
**Clinical Factors**			
Family history of hearing loss (ref: No)			
Yes	1.87	0.90–2.83	<0.001
Weight-loss surgery type (ref: None)			
Bypass surgery	14.37	3.33–25.41	0.011 *
Allergy (ref: No)			
Yes	3.19	2.30–4.07	<0.001
**Model Fit Statistics**			
R^2^	0.143		
Adjusted R^2^	0.118		
F-statistic	5.71 (*p* < 0.001)		
Sample size	1124		

* Statistically significant (*p* < 0.05).

## Data Availability

The data presented in this study is available on request from the corresponding author. The data is not publicly available due to ethical restrictions and privacy concerns related to sensitive health information.

## Abbreviations

The following abbreviations are used in this manuscript:

BMI	Body Mass Index
ET	Eustachian Tube
ETD	Eustachian Tube Dysfunction
ETDQ-7	Eustachian Tube Dysfunction Questionnaire-7
GERD	Gastroesophageal Reflux Disease
OME	Otitis Media with Effusion

## Appendix A

**Table A1:** Determinants of ETD: Multiple linear regression analysis (all variables).

Predictors	β	95% CI	*p*-Value
**(Intercept)**	9.91	6.38–13.43	<0.001
**Anthropometric**			
BMI (per kg/m^2^)	**0.08**	**0.03–0.16**	**0.049**
**Demographic Factors**			
Age (per year)	0.01	−0.04–0.06	0.743
Gender (ref: Female)			
Male	−0.32	−1.35–0.70	0.536
Nationality (ref: Non-Saudi)			
Saudi	−0.05	−1.78–1.68	0.955
**Geographic Factors**			
Region (ref: Central)			
Eastern region	−1.29	−2.89–0.30	0.113
Northern region	−1.17	−2.86–0.52	0.174
Southern region	−1.19	−2.65–0.28	0.112
Western region	−0.70	−2.05–0.65	0.309
Residence (ref: Rural)			
Urban	−0.47	−1.91–0.98	0.525
Nature of residence (ref: Plain)			
Coastal	0.04	−1.06–1.14	0.938
Mountainous	0.64	−0.57–1.85	0.299
**Socioeconomic Factors**			
Education (ref: High school or lower)			
Bachelor’s or Diploma	0.59	−0.40–1.59	0.241
Master’s or PhD	1.29	−0.50–3.09	0.157
Monthly Income (ref: <5000 SAR)			
5000–9999	0.42	−0.85–1.69	0.519
10,000–14,999	0.90	−0.44–2.23	0.190
≥15,000	0.32	−1.08–1.72	0.653
Occupation (ref: Unemployed)			
Employed	−0.40	−1.58–0.78	0.507
Student	0.10	−1.37–1.56	0.898
Marital status (ref: Non-married/Engaged)			
Married	−1.03	−2.39–0.33	0.137
Divorced/Widow	0.62	−1.80–3.04	0.615
**Lifestyle Factors**			
Smoking status (ref: Never)			
Current smoker	−0.65	−2.29–0.99	0.436
Former smoker	−0.08	−2.02–1.86	0.934
Physical activity (ref: None)			
Regular (≥30 min, 5×/week)	−0.28	−1.23–0.67	0.567
Irregular (<30 min, 5×/week)	−0.01	−1.08–1.06	0.983
**Clinical Factors**			
Personal hearing loss (ref: No)			
Yes	1.98	−0.38–4.34	0.101
Family history hearing loss (ref: No)			
Yes	**1.87**	**0.90–2.83**	**<0.001**
Weight-loss surgery type (ref: None)			
Bypass surgery	**14.37**	**3.33–25.41**	**0.011** *
Gastric sleeve surgery	5.57	−4.26–15.41	0.266
Allergy (ref: No)			
Yes	**3.19**	**2.30–4.07**	**<0.001**
**Comorbidities**			
Diabetes Mellitus (ref: No)			
Yes	−0.46	−2.19–1.28	0.606
Hypertension (ref: No)			
Yes	−0.35	−1.97–1.26	0.667
Heart Disease (ref: No)			
Yes	−0.31	−2.69–2.07	0.800
**Model Fit Statistics**			
R^2^	0.143		
Adjusted R^2^	0.118		
F-statistic	5.71 (*p* < 0.001)		
Sample size	1124		

* Statistically significant (*p* < 0.05).
